# Case Report: Successful repair of traumatic right main bronchial transection under veno-venous ECMO support with postoperative inhaled nitric oxide

**DOI:** 10.3389/fmed.2026.1774344

**Published:** 2026-03-30

**Authors:** Xiang Ding, Haobo Kong, Jingjing Pan, Shufeng Yu, Hua Niu, Dahai Zhao

**Affiliations:** 1 Department of Thoracic Surgery, Anhui Chest Hospital, Hefei, Anhui, China; 2 Department of Respiratory Intensive Care Unit, Anhui Chest Hospital, Hefei, Anhui, China; 3 Department of Pulmonary and Critical Care Medicine, Anhui Chest Hospital, Hefei, Anhui, China

**Keywords:** critical care, inhaled nitric oxide, respiratory failure, traumatic bronchial transection, veno-venous extracorporeal membrane oxygenation

## Abstract

**Background:**

Traumatic bronchial transection is a rare but life-threatening consequence of blunt chest trauma. When severe hypoxemia persists despite intubation and mechanical ventilation, maintaining adequate gas exchange during airway reconstruction becomes a major challenge.

**Case presentation:**

We describe a 52-years-old male who sustained a complete right main bronchus transection after a crush-related traffic accident. Despite intubation and maximal ventilatory support, oxygenation remained critically low. Emergency venovenous extracorporeal membrane oxygenation (VV-ECMO) was initiated by the intensive care unit (ICU) team, enabling safe surgical exposure and successful end-to-end bronchial anastomosis. Inhaled nitric oxide (iNO) was administered postoperatively to improve ventilation-perfusion matching. ECMO was discontinued on postoperative day (POD 2) while the patient remained on mechanical ventilation. Inhaled nitric oxide was successfully weaned on POD 4, followed by tracheostomy on the same day. The patient was liberated from mechanical ventilation on POD 13. He recovered fully and was discharged home.

**Conclusion:**

This case highlights the potential role of VV-ECMO as a bridge to repair in traumatic bronchial transection with refractory hypoxemia and suggests that adjunctive iNO may assist perioperative oxygenation management.

## 1 Introduction

Traumatic airway injuries are rare but among the most lethal forms of thoracic trauma. Main bronchial transection represents the most severe type, typically associated with massive pneumothorax, respiratory failure, and a high mortality rate, particularly when diagnosis is delayed or effective ventilation cannot be maintained (1). In cases of complete bronchial rupture, timely surgical repair is essential; however, conventional endotracheal intubation often fails to provide adequate oxygenation or satisfactory surgical conditions due to severe air leakage and the inability to isolate the injured airway (2).

In recent years, veno-venous ECMO (VV-ECMO) has emerged as an important support strategy when conventional airway management is insufficient. For traumatic tracheobronchial injuries, ECMO maintains oxygenation and ventilation despite major airway disruption. It also permits complete airway collapse for optimal surgical exposure while minimizing the risk of intraoperative hypoxemia (3, 4). In addition, adjunctive therapies such as inhaled nitric oxide (iNO) may help stabilize oxygenation and reduce pulmonary vascular resistance during the peri-ECMO period, further supporting respiratory function when the native airway is compromised.

Reports describing ECMO-assisted repair of traumatic main bronchial rupture remain limited. Here, we present a case of a middle-aged man who sustained a complete right main bronchus transection after crush injury. Despite endotracheal intubation, the patient remained hypoxic, prompting initiation of VV-ECMO as a bridge to surgery. Under ECMO support, he successfully underwent end-to-end bronchial anastomosis. Postoperatively, iNO therapy was administered to improve oxygenation. The patient subsequently achieved full recovery and was discharged. This case underscores the essential role of ECMO, complemented by iNO, in ensuring a safe operative field and stable gas exchange during airway reconstruction for severe thoracic trauma.

## 2 Case report

In October 2025, a 52-years-old male construction worker was struck by a vehicle during work, sustaining injuries. Immediately after the accident, the patient experienced severe chest pain accompanied by chest tightness and difficulty breathing. One hour after the incident, the patient was transported to the emergency department of Anhui Provincial Chest Hospital.

**FIGURE 1:**
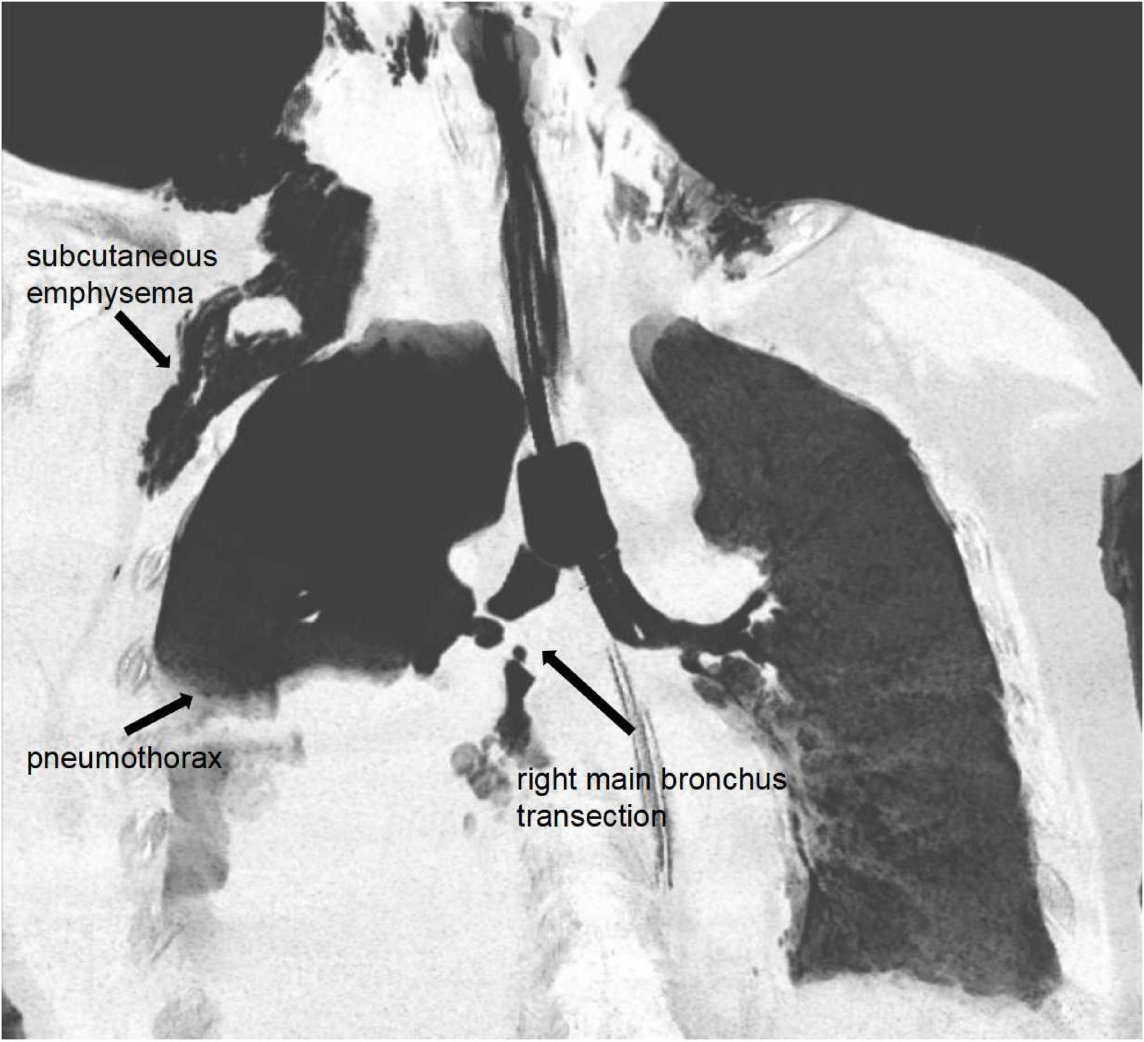
Chest computed tomography revealing complete transection of the right main bronchus with associated pneumothorax and mediastinal emphysema.

Upon admission, the patient was alert but experiencing dyspnea with low oxygen saturation, blood oxygen saturation below 80%. Sedation and analgesia were administered, followed by endotracheal intubation and mechanical ventilation. Physical examination revealed neck swelling with ecchymosis, extensive subcutaneous emphysema visible on the right chest wall, tympanic percussion sounds over the right lung, and absent breath sounds on the right side during auscultation. CT findings demonstrated rupture of the right main bronchus, obstruction by a sputum plug within the right main bronchus, right pneumothorax with atelectasis of the right lung, bilateral subcutaneous emphysema at the cervical roots, chest and abdominal wall, and mediastinal emphysema (Figure 1). A right scapular fracture and multiple bilateral rib fractures were also identified. Preliminary diagnosis: (1) Traumatic bronchial rupture (2) Traumatic hemothorax and pneumothorax (3) Pulmonary contusion (4) Multiple rib fractures in multiple locations (5) Respiratory failure. Following consultation with our hospital’s multidisciplinary team (MDT), it was decided to perform right main airway repair to improve the patient’s ventilation and oxygenation. The patient was on single-lumen endotracheal tube ventilation with left-lung ventilation. The ventilator was set to volume-controlled mode with a tidal volume of 320 ml and a fraction of inspired oxygen of 100%. The patient’s PaO2 (arterial oxygen partial pressure)/FiO2 was 56 mmHg, with a partial pressure of carbon dioxide (PaCO2) at 74.3 mmHg. After comprehensive consideration of the patient’s condition and consultation with the thoracic surgery team, it was decided to perform a tracheal rupture repair under ECMO support.

Veno-venous ECMO was performed via the right femoral vein and right internal jugular vein. Intravenous heparin was administered prior to catheter placement. Under ultrasound guidance, a 21Fr introducer sheath was placed through the right femoral vein using the Seldinger technique, and a 17Fr loop catheter was advanced through the right internal jugular vein at the puncture site. Catheter positioning was adjusted using transabdominal ultrasound. The ECMO circuit utilized a Rotaflow centrifugal pump and a Quadrox PLS oxygenator (Getinge/Maquet Cardiopulmonary GmbH), and the circuit was primed with a balanced electrolyte crystalloid solution according to ECMO protocol. Following priming, the catheter was connected to the patient. The ECMO settings are as follows: initial rotational speed of 3500 rpm, oxygen flow rate of 4 L/min, and blood flow rate of 3.5 L/min. Under this support mode, blood oxygen saturation is maintained at 96%. Intraoperatively, the distal stump of the right upper lobe bronchus was found to be severely crushed and necrotic, with tissue fragmentation extending distally to the segmental bronchial level. The right upper lobe parenchyma was markedly consolidated and non-viable, with no bronchial tissue suitable for reconstruction.

**FIGURE 2:**
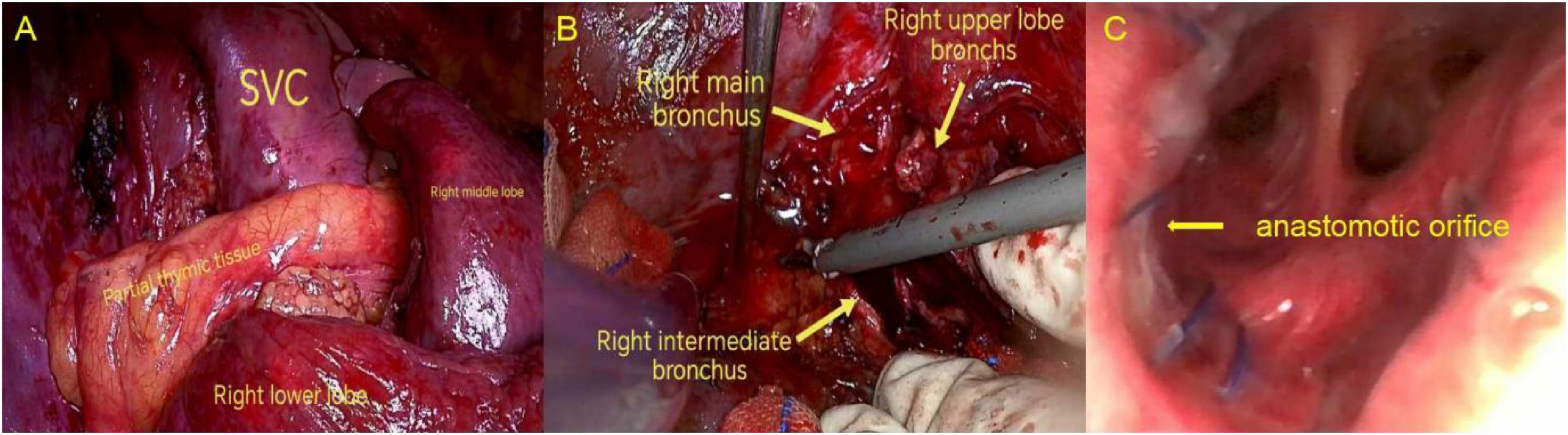
**(A)** Pedicled anterior mediastinal thymic flap wrapped around the bronchial anastomosis for reinforcement. **(B)** Transection of the right main bronchus, bronchus intermedius, and right upper lobe bronchus noted intraoperatively. **(C)** Bronchoscopic examination demonstrated satisfactory healing of the anastomosis. SVC, Superior Vena Cava.

After meticulous debridement of necrotic tissue at the stumps of the right main bronchus and the bronchus intermedius, a tension-free end-to-end anastomosis was performed between the right main bronchus and the bronchus intermedius. Given the extensive bronchial necrosis and irreversible parenchymal consolidation, a right upper lobectomy was subsequently carried out (Figures 2A, B).

Following surgery, the patient was transferred to the intensive care unit and initiated on heparin anticoagulation therapy. Continuous intravenous heparin was administered during VV-ECMO. Given the patient’s major thoracic trauma and fresh bronchial anastomosis, we adopted a low-intensity heparin strategy (target ACT 120–140 s) to mitigate bleeding risk. Circuit function and oxygenator performance were closely monitored, and no oxygenator failure, circuit thrombosis, or clinical thromboembolic events occurred during the short VV-ECMO run. Anticoagulation was additionally monitored using activated partial thromboplastin time (APTT). The ventilator mode was adjusted to pressure-controlled with a target inspiratory pressure of 16 cmH2O, positive end-expiratory pressure (PEEP) of 4 cmH2O, peak inspiratory pressure (PIP) of 13 cmH2O, and a fraction of inspired oxygen of 80%.

On the first postoperative day, a formal assessment for ECMO weaning was conducted. Hemodynamic status remained stable without escalation of vasopressor support. Transthoracic echocardiography demonstrated preserved biventricular function, with a left ventricular ejection fraction of 63% and a tricuspid annular plane systolic excursion (TAPSE) of 21 mm. No right ventricular dilation or dysfunction was observed, and the estimated systolic pulmonary artery pressure was 34 mmHg (TRPG 29 mmHg), indicating the absence of significant pulmonary hypertension.

Despite stable circulatory parameters, oxygenation remained suboptimal, with a PaO2/FiO2 ratio of approximately 150 mmHg, which was considered inadequate for safe discontinuation of sweep gas flow. Inhaled nitric oxide (iNO) at 20 ppm was initiated to improve pulmonary ventilation–perfusion matching. Following initiation of inhaled nitric oxide, the PaO2/FiO2 ratio improved to 208 mmHg. A subsequent sweep gas–off trial demonstrated stable oxygenation without deterioration. ECMO was successfully discontinued on POD 2, and the patient remained on invasive mechanical ventilation. No ECMO-related complications, including bleeding, thrombosis, limb ischemia, or cannulation-site infection, were observed during support or after decannulation.

**FIGURE 3:**
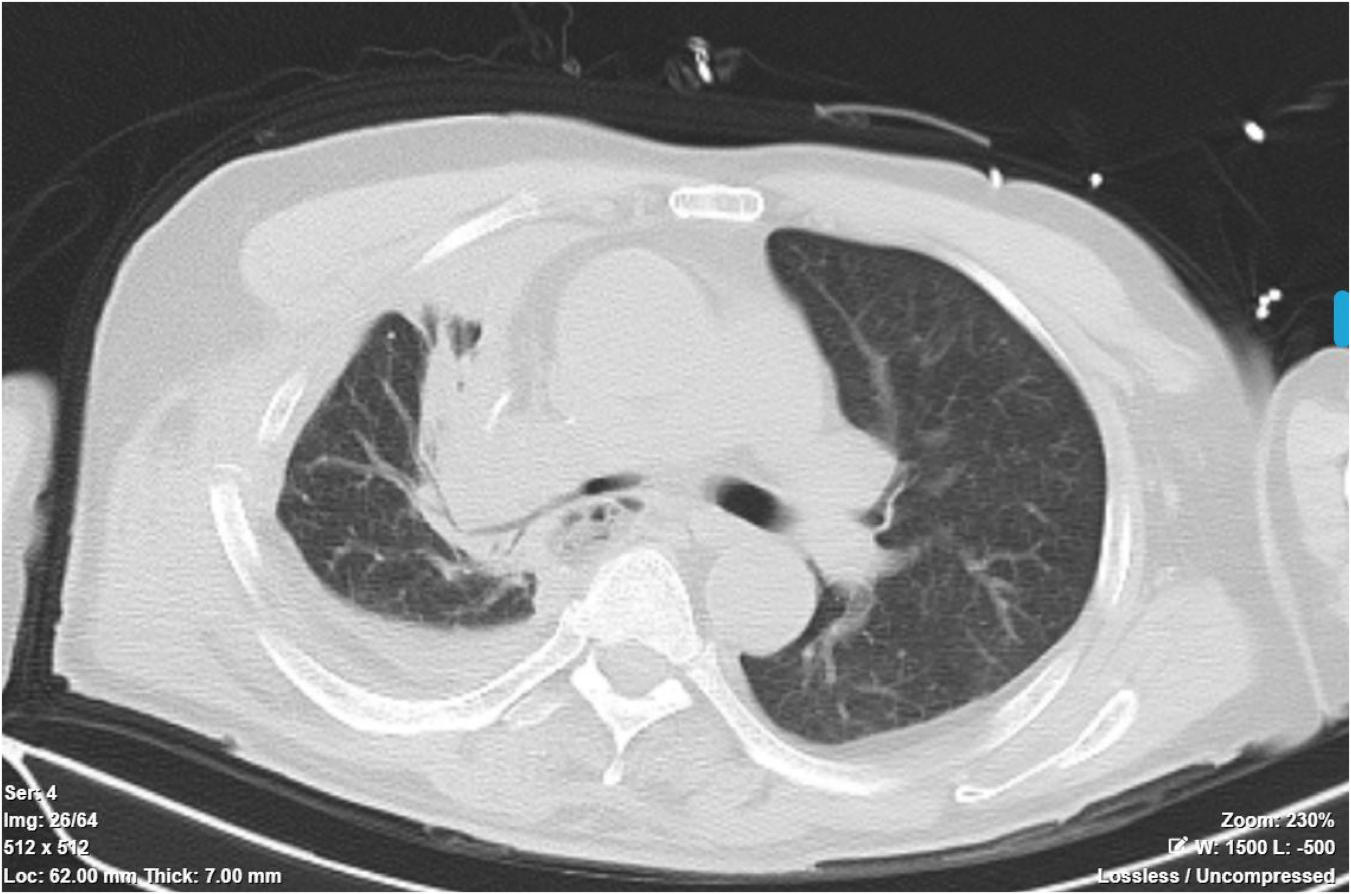
Follow-up chest CT showing lung re-expansion and resolution of pneumothorax after bronchial repair.

**FIGURE 4:**
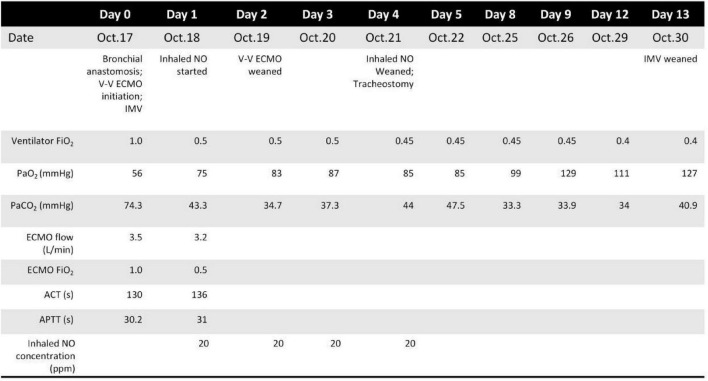
Timeline of clinical course and respiratory support from admission to discharge.

On postoperative day 4, as the patient’s oxygenation had stabilized, inhaled nitric oxide was gradually weaned and ultimately discontinued. The tapering process involved stepwise reduction from 20 to 10 ppm, then to 5 ppm, with close monitoring of arterial blood gases and SpO2 to ensure maintenance of adequate oxygenation. Given the patient’s inadequate recovery of cough reflex, a percutaneous tracheostomy was subsequently performed to facilitate airway management, enabling effective airway clearance, sputum drainage, and bronchoscopic evaluation of the anastomosis. Postoperative bronchoscopy revealed a well-healed anastomotic site (Figure 2C). On postoperative day 13, the patient was successfully weaned from mechanical ventilation. After a brief period of recovery, the tracheostomy tube was occluded. The patient was discharged home for continued rehabilitation. At follow-up, he remained alive, functionally independent, and without evidence of airway stenosis or respiratory insufficiency (Figures 3, 4).

## 3 Discussion

Traumatic main bronchial transection is an uncommon but life-threatening form of blunt chest trauma. Patients frequently present with tension pneumothorax, massive air leak, mediastinal emphysema, and refractory hypoxemia. Delayed diagnosis or inadequate airway control remains strongly associated with mortality (5, 6). Recent studies emphasize that bronchial disruption often cannot be stabilized using conventional endotracheal intubation, as effective isolation of the injured airway is difficult (7, 8). In the published literature, ECMO has increasingly been reported as an adjunct in traumatic tracheobronchial injuries when conventional ventilation is insufficient or when airway reconstruction requires prolonged airway opening with intermittent apnea (9). VV-ECMO provides a safe and effective bridge strategy, maintaining adequate gas exchange while offering optimal surgical exposure for complex airway interventions (10). Xu et al. reported three cases of traumatic bronchial rupture repaired under VV-ECMO support, in which extracorporeal gas exchange provided physiologic stability and an unobstructed operative field, enabling definitive reconstruction and favorable postoperative recovery without major airway stenosis (3). Similar “bridge-to-repair” strategies have also been described in traumatic main airway rupture, where ECMO was instituted prior to surgical repair specifically to prevent catastrophic hypoxemia during airway manipulation (11). In addition, case-level evidence in traumatic tracheal injury supports that VV-ECMO can be used either as rescue therapy during airway compromise or as a planned adjunct to facilitate surgical management (12).

Postoperatively, individualized respiratory support remained crucial. After ECMO weaning on POD2, continued mechanical ventilation was maintained to protect the anastomosis and ensure adequate secretion clearance (3, 7). Excessive coughing, dyssynchrony, or premature extubation may induce anastomotic tension and increase leak risk. Therefore, on POD4, tracheostomy was performed to optimize airway management, reduce sedation requirements, facilitate pulmonary toilet, and enable bronchoscopic reassessment (12, 13). Compared with these reports, our case shares the key principle that VV-ECMO can stabilize gas exchange and allow meticulous airway reconstruction. However, our patient also had substantial lung parenchymal injury and postoperative V/Q mismatch, which likely contributed to delayed oxygenation recovery during weaning assessment. This comparison highlights that the “need for ECMO” in traumatic airway injury is not solely determined by the airway lesion itself, but also by concomitant pulmonary injury and the anticipated inability to maintain adequate oxygenation/ventilation during and immediately after repair. The timing of ECMO initiation is critical. Preoperative cannulation offers greater physiologic stability in patients with severe gas-exchange impairment or anticipated airway compromise, whereas intraoperative initiation reduces ECMO exposure but risks delayed rescue in case of sudden deterioration. In our patient, given the preoperative hemodynamic instability and high risk of ventilation failure during airway manipulation, ECMO was initiated preoperatively to ensure physiologic stability and facilitate a safe and controlled surgical repair.

A notable aspect of this case was the adjunctive use of iNO. On postoperative day 1, following reduction of ECMO oxygenation and sweep gas settings, oxygenation remained inadequate, leading to initiation of iNO at 20 ppm.

As a selective pulmonary vasodilator, iNO preferentially reduces pulmonary vascular resistance in ventilated lung regions, thereby improving ventilation–perfusion (V/Q) matching and decreasing intrapulmonary shunting (14, 15). A case report demonstrates that in critically ill patients receiving VV-ECMO, inhaled nitric oxide (iNO) significantly reduces recirculation in VV-ECMO, improves right ventricular function, and temporarily elevates oxygenation levels (16). These findings support the hypothesis that selective pulmonary vasodilation redistributes blood flow toward better-ventilated regions, thereby improving effective pulmonary blood flow and right heart unloading. Clinically, iNO is frequently used in severe ARDS to transiently improve the PaO2/FiO2 ratio and stabilize gas exchange during profound V/Q mismatch, potentially facilitating ECMO weaning strategies (17). Although inhaled NO is recommended 24–72 h after onset, studies have shown that it is effective when administered early (18), however, a study demonstrated that in ARDS patients treated with VV-ECMO combined with iNO, iNO did not significantly improve the final survival rate (19). In our case, early iNO administration was associated with improved oxygenation during ECMO parameter adjustment. Nevertheless, whether iNO provides sustained clinical benefit in ventilation–perfusion mismatch secondary to traumatic bronchial rupture remains unclear and requires validation in larger, controlled studies.

In addition, the role of tracheotomy in this case also has multiple benefits, which is conducive to long-term clearance of secretions in the airway, reduces the need for sedation to facilitate early awakening for respiratory function exercises, and facilitates tracheoscopy to assess the postoperative anastomosis (20). Several studies suggest that early tracheostomy may improve short-term outcomes in trauma patients with multiple rib fractures. In a propensity score–matched analysis, early tracheostomy was associated with significantly improved 28-days survival compared with delayed intervention (21).

This case highlights the critical role of the intensive care unit in managing complex airway trauma. When conventional ventilation methods prove inadequate, the emergency activation of ECMO therapy can potentially save lives (6, 22). This report represents a single case, which limits the generalizability of the findings. Moreover, the improvement in oxygenation observed after inhaled nitric oxide administration may have been partially attributable to postoperative stabilization and ECMO weaning, preventing definitive conclusions regarding causality. Further studies with larger cohorts are warranted to better define the role of adjunctive iNO in traumatic airway injury supported by VV-ECMO.

## 4 Conclusion

ECMO provides essential respiratory support and ensures safe surgical conditions for patients with severe tracheobronchial injuries during the perioperative period. Nitric oxide inhalation therapy optimizes postoperative gas exchange in these patients. The combination of both approaches may facilitate early recovery and optimize perioperative respiratory management in selected patients, suggesting that this strategy may be considered in selected cases of traumatic bronchial injury.

## Data Availability

The original contributions presented in this study are included in this article/supplementary material, further inquiries can be directed to the corresponding authors.
